# Overexpression of TELO2 decreases survival in human high-grade gliomas

**DOI:** 10.18632/oncotarget.10021

**Published:** 2016-06-14

**Authors:** Shao-Wei Feng, Ying Chen, Wen-Chiuan Tsai, Hsin-Ying Clair Chiou, Sheng-Tang Wu, Li-Chun Huang, Chin Lin, Chih-Chuan Hsieh, Yun-Ju Yang, Dueng-Yuan Hueng

**Affiliations:** ^1^ Department of Neurological Surgery, Tri-Service General Hospital, National Defense Medical Center, Taipei, Taiwan, R.O.C.; ^2^ Department of Biology and Anatomy, National Defense Medical Center, Taipei, Taiwan, R.O.C.; ^3^ Department of Pathology, Tri-Service General Hospital, National Defense Medical Center, Taipei, Taiwan, R.O.C.; ^4^ Division of Urology, Department of Surgery, Tri-Service General Hospital, National Defense Medical Center, Taipei, Taiwan, R.O.C.; ^5^ Department of Biochemistry, National Defense Medical Center, Taipei, Taiwan, R.O.C.; ^6^ Graduate Institute of Life Science, National Defense Medical Center, Taipei, Taiwan, R.O.C.; ^7^ Graduate Institute of Medical Science, National Defense Medical Center, Taipei, Taiwan, R.O.C.

**Keywords:** gliomas, WHO grades, GEO profile, Telomere maintenance 2, TELO2

## Abstract

High-grade gliomas are characterized with poor prognosis. To improve the clinical outcome, biomarker is urgently needed for distinguishing oncotarget in high-grade gliomas. *Telomere maintenance 2 (TELO2)* regulates S-phase checkpoint in cell cycle, and is involved in DNA repair. However, the role of *TELO2 in survival outcome of high-grade gliomas is still not yet clarified. This study aims to* investigate the correlation between *TELO2* mRNA expression and survival outcome of patients with high-grade gliomas. Based on bioinformatics study, we found that Kaplan-Meier analysis demonstrated shorter survival in patients with higher *TELO2* mRNA levels than in those with lower *TELO2* expression (median survival, 59 vs. 113 weeks, *p=0.0017*, by log-rank test, hazard ratio: 0.3505, 95% CI: 01824.-0.6735). *TELO2* mRNA expression significantly higher in World Health Organization (WHO) grade IV than in non-tumor control (*p*=2.85 × 10^−9^). Moreover, *TELO2* level was greater in WHO grade III than in non-tumor controls (*p*= 0.017) human gliomas. We further validated *TELO2* mRNA expression and protein levels by using quantitative RT-PCR, Western blot, and immunohistochemical (IHC) stain of tissue microarray. Consistently, the *TELO2* mRNA and protein expression were significantly elevated in human glioma cells in comparison with normal brain control. Additionally, IHC staining showed higher TELO2 immunostain score in high-grade gliomas than in low-grade gliomas, or normal brain control. Taken together, human high-grade gliomas increase *TELO2* mRNA expression, and overexpression of *TELO2* mRNA expression correlates with shorter survival outcome, supporting that *TELO2* is an oncotarget in human gliomas.

## INTRODUCTION

World Health Organization (WHO) grade III anaplastic astrocytoma, and grade IV glioblastoma multiforme (GBM) are defined as high-grade gliomas with poor survival outcome [1, 2]. Conventional therapeutic strategies consist of extensive resection, concurrent chemo-radiotherapy. However, high-grade gliomas still progressed and recurred despite of aggressive therapies [3]. Therefore, biomarker for distinguishing clinical outcome is urgently needed in high-grade gliomas to provide oncotarget for clinician to improve the clinical outcome [4, 5].

*Telomere maintenance 2 (TELO2)* was first found in *Saccharomyces cerevisiae* to regulate the telomere length [6]. Later, *TELO2* was found in *Caenorhabditis elegans* to control biological rhythms and life span and named *CLK2* [7, 8]. *TELO2* located in chromosome 16 and also called *TEL2* [9]. *TELO2* encodes protein for regulation of S-phase checkpoint protein in cell cycle, ataxia telangiectasia and Rad3-related (ATR) signaling pathway, and involves in DNA repair [10], and radiation sensitivity [11]. Studies showed *TEL2/CLK2* orthologs increased the telomere length in *Caenorhabditis elegans* and the budding yeast *Saccharomyces cerevisiae* [12–15].

Overexpression of *TELO2* accelerated cell cycle, induced cells hypersensitive to apoptosis caused by DNA replication block or oxidative stress, and gradually expanded telomere length in human liver adenocarcinoma cell line [9]. However, the correlation of *TELO2* with the human gliomas is not yet elucidated. This study aims to clarify whether *TELO2* expression correlated with survival prognosis and the WHO pathological grading. The Gene Expression Omnibus (GEO) profiles offer database to assess the gene expression of particular disease. Investigating *TELO2* expression level in GEO dataset revealed higher expression and shorter survival outcome in high-grade gliomas than in low-grade gliomas. Validation of *TELO2* using qRT-PCR, Western blot and immunohistochemical (IHC) staining supports the overexpression of *TELO2* mRNA and protein level in high-grade gliomas, implying that *TELO2* correlates with adverse outcome in high-grade gliomas.

## RESULTS

### Demographic data of human high-grade gliomas

The demographic data including gender, age, WHO pathologic grades, and survival times were shown in Table 1, and Table 2. There were total 100 patients contained in WHO grade IV (76), and grade III (24). After exclusion 20, and 3 missing data in WHO grade IV, and III (76, and 24 respectively), remaining 56 patients (18 female, 38 male) in grade IV population, and 21 patients (8 female, 13 male) in grade III population. The age of grade IV, and III patients were 48.52 ± 12.82, and 37.38 ± 9.86 years old, respectively (*P* = 0.001). The overall survival time in grade IV, and III was 106.73 ± 82.02, and 226.95 ± 161.12 weeks, respectively (*P* = 0.003) (Table 1). Moreover, as shown in Table 2, univariable analysis identified three significant predictors (WHO grade, *TELO2* mRNA expression, and age). Multivariable analysis further confirmed WHO grade, and *TELO2* mRNA expression were significant predictors (*P* = 0.0056, and 0.0141, respectively).

**Table 1:** Analyses of demographic data in human high-grade gliomas

	Grade III	Grade IV	*P* value
	n=24	n=76	
Gender			0.968^§^
Male	16(66.7%)	51(67.1%)	
Female	8(33.3%)	25(32.9%)	
Age TELO2 expression	37.38±9.86^a^	48.52±12.82^b^	0.001* 0.024*
< 153.15	22(91.7%)	52(68.4%)	
> 153.15	2(8.3%)	24(31.6%)	
Survival time (weeks)	226.95±161.12^a^	106.73±82.02^b^	0.003*

TELO2: Telomere maintenance 2;

* ***P***<0.05, by Wilcoxon Test;

§ Chi-square analysis

a There are three missing data;

b 20 missing data.

**Table 2:** Statistical analyses of the association between demographic data and survival time using Cox proportional hazard model

	Univariable analysis Crude-HR (95% CI)	*P* value	Multivariable analysis Adj-HR (95% CI)	*P* value
WHO grade				
III	1.000		1.000	
IV	3.223 (1.735 to 5.987)	0.0002*	2.518 (1.310 to 4.840)	0.0056*
*TELO2* mRNA expression				
< 153.15	1.000		1.000	
> 153.15	2.312 (1.350 to 3.959)	0.0023*	2.051 (1.156 to 3.640)	0.0141*
Gender				
Male	1.000		1.000	
Female	0.761 (0.468 to 1.235)	0.2689	0.736 (0.443 to 1.223)	0.2367
Age (per 20 years)	1.587 (1.087 to 2.317)	0.0169*	1.226 (0.832 to 1.809)	0.3032

WHO: World Health Organization; TELO2: Telomere maintenance 2; CI: confidence interval;

* *P*<0.05 with statistical significance

### Overexpression of *TELO2* mRNA expression correlates with poor survival in high-grade gliomas

**Figure 1:**
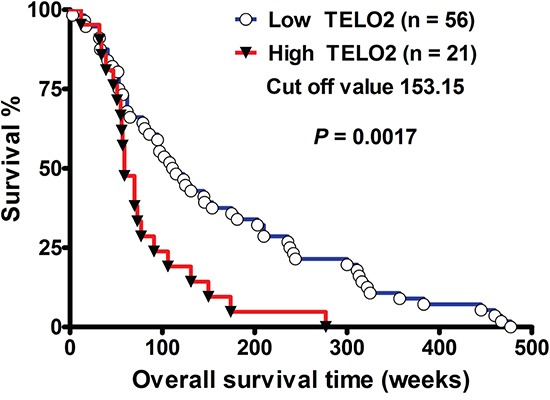
*TELO2* mRNA overexpression decreases survival in human high-grade gliomas The Kaplan-Meier survival curve showed shorter survival in those with high *TELO2* (> 153.15) (n=21) compared to those with low *TELO2* (< 153.15) (n=56) expression levels (median survival, 113 vs. 59 weeks, *P*=0.0017, by log-rank test, hazard ratio: 0.3505, 95% CI: 0.1824-0.6735).

To investigate the role of *TELO2* in human glioma, we first analyzed the correlation of *TELO2* expression with overall survival of human high-grade gliomas. As shown in Figure 1, the Kaplan-Meier survival curve of 77 patients with high-grade gliomas revealed that patients with low *TELO2* mRNA expression levels (n = 56) had longer overall survival than those with high *TELO2* mRNA expression levels (n = 21) (*P* = 0.0017, by log-rank test; 95% CI: 0.1824-0.6735, Ratio 0.3505). The cut-off value was set at 153.15. The median survival interval in the high- and low-*TELO2* expressions level was 59 weeks and 113 weeks, respectively. These data demonstrate that high *TELO2* mRNA expression correlates with shorter survival in human high-grade gliomas, supporting the hypothesis of *TELO2* belongs to oncogene.

### Human high-grade gliomas increase *TELO2* mRNA expression

**Figure 2:**
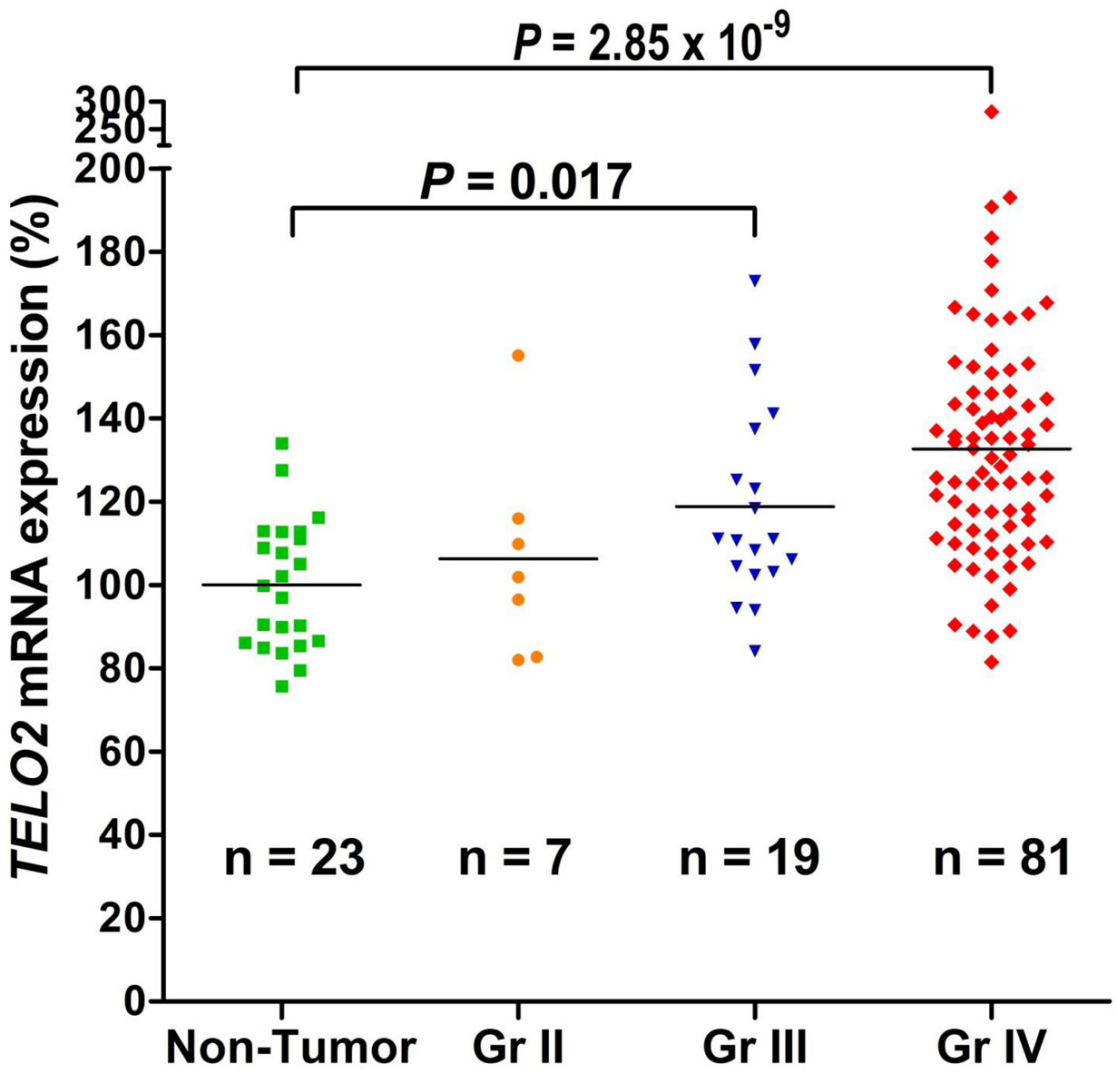
Human high-grade gliomas increase *TELO2* mRNA expression The scattered plots showed the *TELO2* mRNA gene expression in WHO grade II, III, IV, and non-tumor control. Increased *TELO2* mRNA levels significantly correlated with WHO grades of human high-grade gliomas. The Y-axis was TELO2 mRNA expression (%). The original mean value of non-tumor group was 292.7. The non-tumor group represented the baseline (100%). The *P* value was adjusted using Bonferroni method between each group.

Next, we analyzed the correlation of *TELO2* mRNA expression and pathological grading. As shown in Figure 2, *TELO2* mRNA expression level was statistically greater in WHO grade IV (n = 81) then in non-tumor controls (n = 23) (*P* = 2.85 × 10^−9^). Moreover, the *TELO2* mRNA level was also significantly higher in WHO grade III (n = 19) than in non-tumor controls (*P =* 0.017, *P* adjusted by Bonferroni method). Consistently, these data further support the hypothesis of *TELO2* belonging to oncogene in human high-grade gliomas.

### Validation the level of *TELO2* mRNA expression in human glioma cell lines

**Figure 3:**
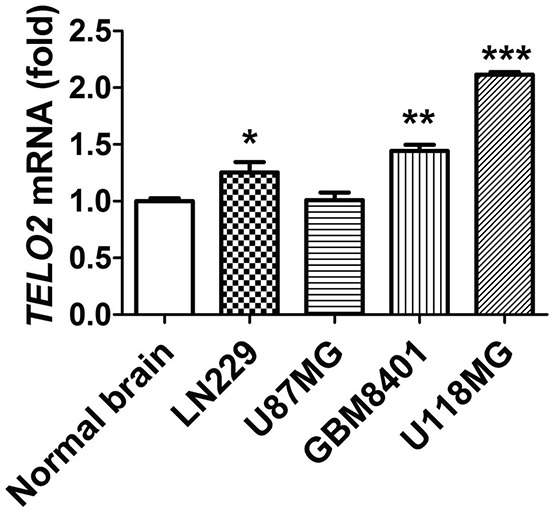
Validation of TELO2 mRNA expression in glioma cell lines and normal brain tissue qRT-PCR was conducted to examine *TELO2* mRNA expression and the quantitative results were shown. The relative expressions were normalized with normal brain. Bars, mean±SEM, *P < 0.05, **P < 0.01, ***P < 0.005 showed significant differences. Data are representative of three independent experiments.

Since two dry lab datasets of quantitative gene study supporting the role of *TELO2* is oncogene in human high-grade gliomas. We further validate the level of *TELO2* mRNA expression in human normal brain and three glioma cell lines, LN229, GBM8401, and U118MG through wet lab approach using q-RT PCR. As shown in Figure 3, the mRNA expression of *TELO2* was significantly higher in human LN229 glioma cell line than in normal brain (*P* < 0.05). Moreover, *TELO2* was also statistically higher in human GBM8401 and U118MG glioma cell line than in normal brain (P < 0.01, P < 0.005, respectively). These data further consolidate the high expression levels of *TELO2* mRNA in human gliomas.

### TELO2 hub protein and the protein-protein interactions

**Figure 4:**
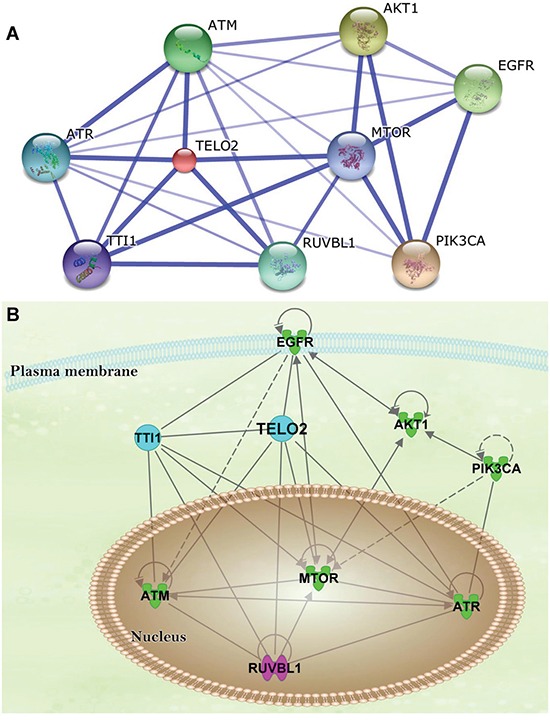
The protein-protein interaction (PPI) network and ingenuity pathway analysis (IPA) **A**. The PPI network established by the STRING database. TELO2 is controlling hub; and **B**. The IPA predicted the mTOR-regulated AKT1, EGFR, and PIK3CA signaling pathways transcriptionally regulated through TELO2.

**Figure 5:**
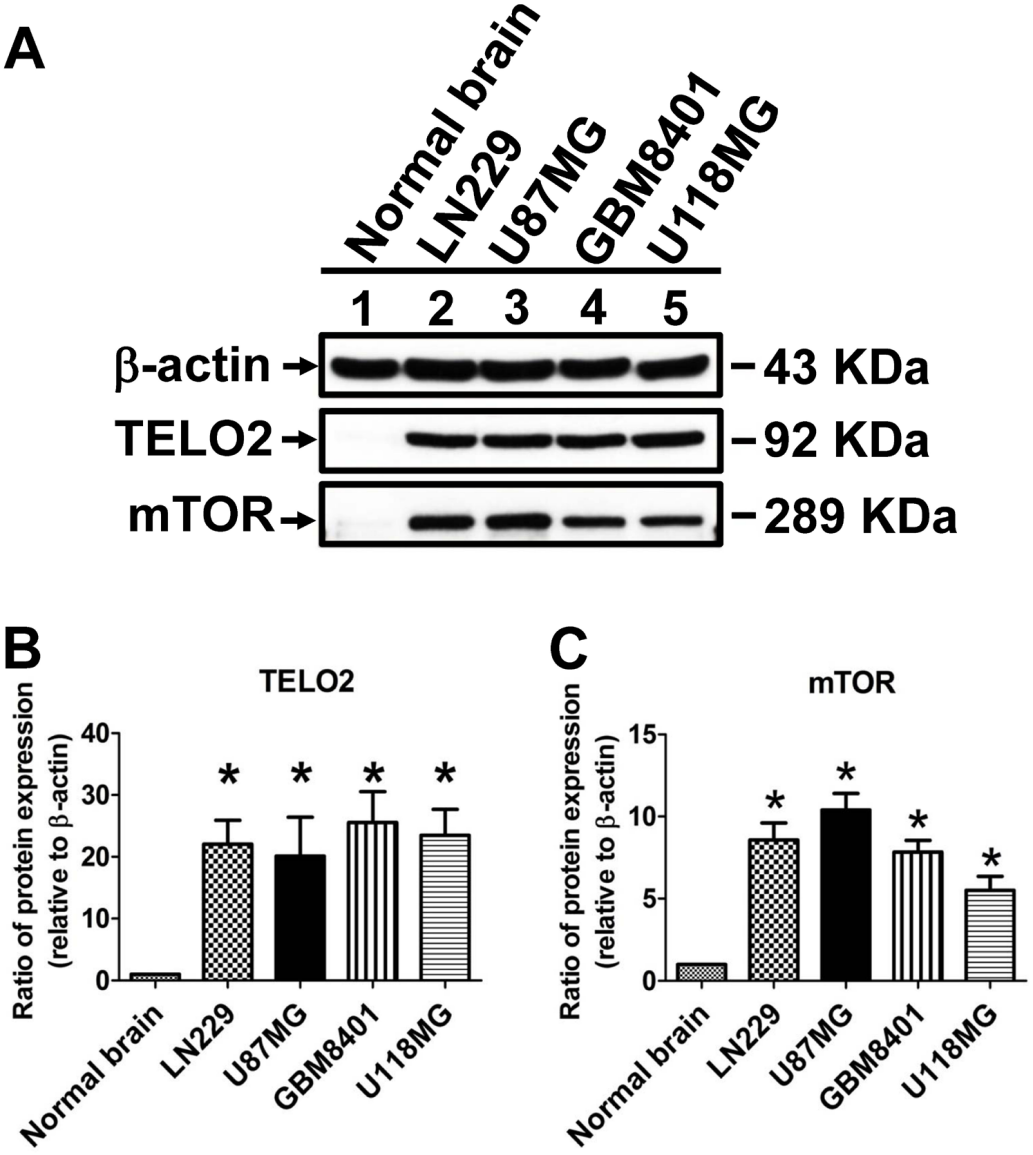
Validation and statistical analyses of TELO2 and mTOR protein levels **A.** Protein lysates of glioma cell lines, including LN229, U87MG, GBM8401 and U118MG, were applied to SDS-PAGE and Western blotting. The statistical analyses of TELO2 and mTOR protein levels were presented in **B** and **C.** β-actin served as a loading control. Bars, mean±SEM, *P < 0.05 showed significant differences in comparison with normal brain control.

The protein-protein interaction (PPI) network of TELO2-regulated oncogenesis was created using Search Tool for the Retrieval of Interacting Genes/Proteins (STRING) database. The network showed TELO2 is a hub protein regulating the signaling pathway among the mammalian target of rapamycin (mTOR), TELO2 interacting protein 1 (TTI1), ataxia telangiectasia and Rad3-related (ATR), ataxia telangiectasia-mutated (ATM), and RuvB-like 1 (RUVBL1) (Figure 4A). Moreover, the Ingenuity pathway analysis (IPA) predicts that TELO2 plays the transcriptional regulation role for ATM, mTOR, ATR and RUVBL1 to translocate into nucleus in signaling pathways (Figure 4B). Finally, the protein production of TELO2 and mTOR in human normal brain and four glioma cell lines, LN229, U87MG, GBM8401, and U118MG through wet lab approach using Western blotting (Figure 5A). The protein level of TELO2 significantly increased correlates with the mTOR expression in human glioma cell lines including U118MG, GBM8401, U87MG, and LN229 as compared with normal brain tissue (Figure 5B and 5C). Taken together, these studies demonstrated the expression of TELO2 correlated with mTOR expression in human gliomas.

### TELO2 protein production overexpresses in human high-grade gliomas

**Figure 6:**
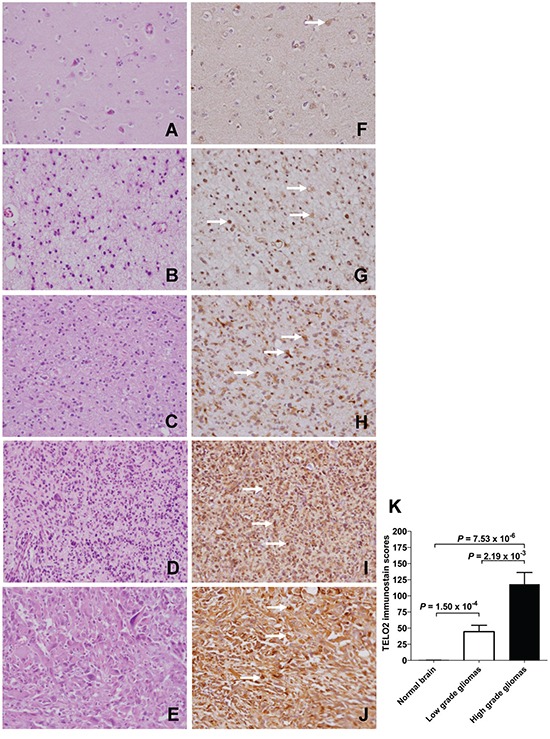
Overexpression of TELO2 in high-grade gliomas compared to low-grade gliomas and non-tumor control Representative hematoxylin and eosin staining of **A.** normal brain, **B.** pilocytic astrocytomas, **C.** diffuse astrocytomas, **D.** anaplastic astrocytomas, and **E.** glioblastomas multiforme. Representative immunohistochemical staining of TELO2 in **F.** normal brain, **G.** pilocytic astrocytomas, **H.** diffuse astrocytomas, **I.** anaplastic astrocytomas, and **J.** glioblastomas multiforme. **K.** Statistical analysis showed the TELO2 immunostain score was significantly higher in high-grade (WHO grade III, and IV) gliomas than in in low-grade (WHO grade I, and II) gliomas, and normal brain (*P* = 2.19×10^−3^, 7.53×10^−6^, respectively), Moreover, the TELO2 immunostain score was significantly higher in low-grade gliomas than in normal brain control (*P* = 1.54×10^−4^, *P* adjusted by Bonferroni method). The significant expression of TELO2 in glioma cell was labeled by arrow marks. Original magnification × 400.

To investigate the TELO2 protein production in human gliomas tissues and normal brain tissues, IHC staining of human tissue microarray was conducted (Figure 6A to 6J). The TELO2 immunostain score was significant higher in high-grade (WHO grade III, and IV) gliomas than in low-grade (WHO grade I, and II) gliomas or normal brain (*P* = 2.19×10^−3^, 7.53×10^−6^, respectively). Moreover, TELO2 immunostain score was significant higher in low-grade gliomas than in normal brain (P = 1.54×10^−4^, P adjusted by Bonferroni method, Figure 6K), supporting TELO2 protein overexpressed in high-grade gliomas in comparison with normal brain tissue control.

## DISCUSSION

This study showed that high *TELO2* mRNA expression predicts poor survival outcome in comparison with low *TELO2* mRNA expression. Moreover, *TELO2* expression was significantly greater in patients with high-grade gliomas than in low-grade gliomas and in those with non-tumor brain controls. Validation using qRT-PCR, Western blotting, and IHC staining further confirmed that *TELO2* overexpresses in high-grade gliomas. To our understanding, this is the first study to launch the correlation of *TELO2* biomarker with WHO pathological grading in human gliomas, and survival outcome, supporting *TELO2* belongs to oncogene.

TELO2 as an essential orphan protein involved in many function including DNA repair, telomere maintenance [13], the biological clock, signal transduction pathways, and DNA damage checkpoints [11, 16–18]. Treatment of *TELO2* (also called *CLK2*) small interference RNA significantly decreased growth rate and arrested the cell cycle reversibly of human hepatic adenocarcinoma cell. In contrast, overexpression of *TELO2* increased the growth rate of SK-HEP-1 cells through the mechanisms of shortened cell cycle length, and gradually expanded telomere length in human SK-HEP-1 liver adenocarcinoma cell line [9], suggesting the role of *TELO2* for glioblastoma growth. Both genomic and proteomic investigations showed that there is virtually common activation of the PI3K/Akt/mTOR signaling pathway in human glioblastomas [1, 19–21]. PI3K signaling increases glioblastomas formation and tumor progression in mouse-engineered models, launching PI3K and its effecter mTOR as convincing targets. mTOR incorporates oncogenic signaling from some growth factor receptors via PI3K, to modulate nutrient status and cellular energy, to stimulate downstream targets that promote tumor growth and suppress glioma cell invasion [22, 23]. The standard chemotherapy for GBM is temozolamide (TMZ) [24], an alkylation agent that leads to DNA damage. However, the therapeutic effect of TMZ will decrease, and the drug resistance occurs due to the activation of PI3K/Akt/mTOR signaling pathway. Tel2 and Tti1 stabilization correlated with the elevated expression levels of all phosphatidylinositol 3-kinase related protein kinase (PIKK) family. This effect was most obvious in mTOR [25]. Consistently, our data further showed higher expression of TELO2 correlated with the poor survival outcome in high-grade gliomas, supporting the oncogenic role of TELO2.

We applied STRING to identify the PPI [26]. STRING platform found some important proteins were linked by hub protein TELO2, and mTOR. PPI predicts the TELO2 protein is involved in transcriptional regulation of the mTOR, RUVBL1, ATR, and ATM in signaling pathways. Moreover, mTOR also plays a hub protein of TELO2, ATM, AKT1, EGFR, PIK3CA, and RUVBL1 in signaling pathways. TELO2 is required for the stable expression of all six members of the PIKK family, including ATM, ATR, DNA-dependent protein kinase catalytic subunit (DNA-PKcs), and mTOR [27]. Importantly, mTOR inhibition related to feedback activation of PI3K/Akt signaling and regulation of other crucial cellular signals in human glioblastoma to escape from the effect of mTOR inhibitor rapamycin [20]. TELO2, TTI1 and TTI2 (Tel two-interacting protein 2) compose a 2-MDA complex called the Triple T complex. DNA damages response (DDR) is critical for maintain genomic stability, which is a complex signal transduction network including activated transcription and DNA repair, and coordinated cell cycle transitions. ATM and ATR are central regulators of the DDR. The triple T complex was noted required for DNA damage single, and stabilize the ATM and ATR [10]. In addition, depletion of TELO2 would decrease the mTOR protein level but not the mRNA level through the binding of R2TP Complex to TELO2 which phosphorylated by casein kinase 2 (CK2) [27, 28]. These studies suggested that the *TELO2* gene could be the novel therapeutic targets for high-grade gliomas. Further study of downstream targets (pS6k, 4EBP1, pp70S6k) in signaling pathways activated in glioma cell lines would be another one interesting study in the future. The main theme of this study is to clarify the novel role of TELO2 expression in clinical significance of high-grade glioma patients. Further statistical analysis of patient clinical samples in serial histological sections would explore a novel discovery between mTOR and TELO2 in future study.

The limitation of this study included difficulty in collecting massive amounts of human brain gliomas, grade I low-grade glioma samples are rare, and paired non-tumor control for validation of protein level or mRNA expression. Alternatively, large-scale analyses of total 280 sheets of data from two GEO profiles were conducted to determine the role of *TELO2* as a pathological grade and survival outcome marker. Since the gene expression profilings were quite different between tissue and cell lines, therefore, low grade compared with high grade glioma cell lines would be a better experimental design. Alternatively, the normal cell line could be as a reference.

In conclusion, human high-grade gliomas increase *TELO2* mRNA expression. Overexpression of *TELO2* mRNA expression correlates with shorter survival outcome. Validation of gene and protein levels, using qRT-PCR, Western blotting, and IHC staining, consistently supports that *TELO2* is an oncotarget in human gliomas.

## MATERIALS AND METHODS

### Analysis of human gliomas datasets from GEO profile

This study was approved by the institutional review board (TSGHIRB No: B-102-10) of Tri-Service General Hospital in Taipei, Taiwan, ROC. The genomic databases obtained from the GEO profile were analyzed by previous methodology [29, 30, 32]. Briefly, 100 sheets of de-linked data (GDS1815 / 209528_s_at / TELO2) on *TELO2* mRNA expression, age, gender, overall survival time, and pathologic grading of primary high-grade glioma were gathered from http://www.ncbi.nlm.nih.gov/geo/tools/profileGraph.cgi?ID=GDS1815:209528_s_at. Twenty-three sheets of data without thorough information on survival period and age were excluded so that 77 sheet were collected in the statistical analyses.

The analysis of *TELO2* gene expression and pathological grading obtained from additional database GDS1962 / 34260_s_at / TELO2 including 180 sheets from 81 patients with grade IV glioma, 19 with grade III glioma, seven with grade II glioma, 23 without tumor (non-tumor control) searching from http://www.ncbi.nlm.nih.gov/geo/tools/profileGraph.cgi?ID=GDS1962:34260_at.

### RNA isolation and cDNA synthesis

Total RNA of glioma cell lines were extracted using the EasyPure Total RNA reagent (Bioman, Taiwan, ROC) according to the manufacturer's protocol. Briefly, cells were harvested and lysed in EasyPure Total RNA reagent. After the addition of chloroform followed by centrifugation, the aqueous phase were transferred into a new tube for RNA recovery by precipitation with isopropyl alcohol. cDNA were prepared using oligo dT, and MMLV Reverse transcriptase (Epicentre Biotechnologies, USA). The normal brain cDNA was purchased from Origene Technologies (Rockville, MD, USA).

### Quantitative real-time PCR (qRT-PCR)

qRT-PCR was carried out in a 20 μl volume using IQ^2^ fast qPCR system with ROX (Bio-genesis Technology Inc., Taipei, Taiwan) in illumine ECO™ Real-Time PCR system. The relative gene expression was analyzed using the 2^−ΔΔCt^ method against GAPDH expression. PCR primers for each gene were obtained from PrimerBank (http://pga.mgh.harvard.edu/primerbank/links.html). The primer pairs of *TELO2* and *GAPDH* were summarized in Table 3.

**Table 3:** Sequences of primers used in polymerase chain reaction

Gene	Oligonucleotide	Sequence (5′-3′)	Product Size (bp)
TELO2	Forward	CCCGCAGAGATCGTGGATG	96 bp
	Reverse	CATGTCGTAGGGGACAAACTC	
GAPDH	Forward	GCACCGTCAAGGCTGAGAAC	142 bp
	Reverse	ATGGTGGTGAAGACGCCAGT	

### Cell lysate preparation and western blotting

Cells were lysed by RIPA buffer (100 mM Tris-HCl, 150 mM NaCl, 0.1% SDS, and 1% Triton-X-100) and the cell lysates were harvested by centrifugation at 15,000 rpm for 10 min to remove the debris. The normal brain lysate were purchased from Origene Technologies. Thirty-microgram cell lysates from each group were applied to 10% sodium dodecyl sulfate polyacrylamide gels electrophoresis and proteins were transferred onto polyvinyl difluoride membranes (Millipore, MA, USA). The membrane was blocked with 5% skim milk in TBST for 1 h at room temperature. The antibodies used include anti-TELO2 (Cat. No. ab122722, Abcam, Cambridge, UK), mTOR antibody (cat. No. 2972; Cell Signaling Technology, Beverly, Boston, MA, USA), and β-actin (Santa Cruz Biotechnology, Inc.). Band detection was conducted by enhanced chemi-luminescence and X-ray film (GE Healthcare, Piscataway, NJ, USA).

### Protein-protein interactions network analysis

Search Tool for the Retrieval of Interacting Genes/Proteins (STRING) database (http://string-db.org) was applied for analysis of predicted and known PPI [26]. PPI of TELO2 was performed according to the method of our previous publication [29].

### Immunohistochemical staining of human gliomas specimen

IHC staining of commercially available tissue microarray (BS17016; Biomax, Rochester, NY, USA) was performed according to previous protocol [29–31], incubated with a polyclonal rabbit anti-human TELO2 antibody (Cat. No. ab122722, Abcam, Cambridge, UK) (1:100 diluted in phosphate buffered saline (PBS) for 1 h at room temperature, washed 3 times (each for 5 min in PBS), incubated with biotin-labeled secondary immunoglobulin (1:100, DAKO, Glostrup, Denmark) for 1 h at room temperature, washed 3 times, and treated with 3-amino-9-ethylcarbazole substrate chromogen (DAKO) at room temperature to visualize peroxidase activity [32]. Sections of human urinary bladder cancer tissue (known to stain positive for TELO2) were used as positive control according to the datasheet of TELO2 antibody (ab122722).

### Statistical analysis

The methodology of statistical analysis has been defined proceeding [17, 30]. Two datasets (GDS1815 / 209528_s_at / TELO2 and GDS1962 / 34260_s_at / TELO2) gather from the GEO profiles were analyzed. The single tail test was used to calculate the *TELO2* expression level in four groups of WHO pathologic grades gilomas. The Bonferroni method was used to adjust the *P* value to avoid the potentiality of type I error in multi-groups analyses. Overall survival was analyzed by The Kaplan-Meier method. Cohorts of low-versus high-*TELO2* gene expressions were evaluated in WHO grade IV combined with grade III human glioma groups. Chi-square test was applied to analyze survival parameters in patients from high-grade gilomas. The cut-off value of *TELO2* expression is decided based on conditional inference tree via ‘party’ package with R language (R 3.1.2 software). Conditional inference tree is a ‘decision tree’-based method. It can help us to classify patients and reduce the entropy of data. GraphPad Prism 5 software was used to draw figures. The Y-axis was presented as *TELO2* mRNA expression (%). The *TELO2* mRNA expression of each WHO grade glioma showed in percentage to non-tumor group (baseline 100%). The *P* value less than 0.05 reached statistical significance.
